# Density functional theory calculations on graphene/α-SiO_2_(0001) interface

**DOI:** 10.1186/1556-276X-7-158

**Published:** 2012-02-28

**Authors:** Zhimin Ao, Man Jiang, Zi Wen, Sean Li

**Affiliations:** 1 Key Laboratory of Automobile Materials, Ministry of Education and Department of Materials Science and Engineering, Jilin University, Changchun, 130022, China; 2 School of Materials Science and Engineering, The University of New South Wales, Sydney, NSW 2052, Australia; 3 Department of Chemistry, Jilin University, Changchun, 130022, China

## Abstract

In this work, the graphene/α-SiO_2_(0001) interface is calculated using density functional theory. On the oxygen-terminated SiO_2 _surface, atomic structure reconstruction occurs at the graphene/SiO_2 _interface to eliminate the dangling bonds. The interface interaction is 77 meV/C atom, which indicates that van der Waals force dominates the interaction, but it is stronger than the force between the graphene layers in graphite. The distance between graphene and the SiO_2 _surface is 2.805 Å, which is smaller than the 3.4 Å interlayer distance of graphite. In addition, the SiO_2 _substrate induces *p*-type doping in graphene and opens a small gap of 0.13 eV at the Dirac point of graphene, which is desirable for electronic device applications.

## Introduction

Graphene, a single two-dimensional layer of graphite in hexagonal structure, is the starting point for many nanographite devices with promising electrical properties [1]. After the theoretical prediction of the peculiar electronic properties of graphene in 1947 by Wallace [2] and the subsequent studies on its magnetic spectrum [3,4], it took half a century until the graphene could be first experimentally fabricated [1], and its anomalous quantum Hall effect has been measured [5-7], which encourages numerous works on it now [8-11].

However, the instability of a freestanding graphene (it has an intrinsic three-dimensional structure or ripples) [12] leads graphene to be used on devices by laying it on a substrate. For example, the insulating substrates made from silicon dioxide [SiO_2_] are widely used as a dielectric medium in electronic devices. The electrical properties of graphene can be modified using electrical gates, substrates, and chemical species such as atoms and molecules [13,14]. It has been reported that graphene grown on a SiC surface was *n*-type and was exhibiting a gap of about 0.26 eV [15]. A theoretical study on bilayer graphene grown on a SiC surface showed that the first carbon layer formed covalent bonds with SiC and acted as a buffer layer, and the graphene nature properties were recovered by the second carbon layer [16,17]. For graphene supported by SiO_2_, previous works studied the electrical properties of graphene and the doping effect of the SiO_2 _substrate [18-23]. They found that graphene was *p *doped when the graphene was weakly bonded to an O-terminated surface with hydrogen passivation. However, graphene would exhibit a finite bandgap if there is a strong interaction with an O-terminated surface while *n *doping took place on a Si-terminated surface with the active dangling bonds [20,21]. In these works, they considered that graphene would form C-O or C-Si bonds on the interface due to the dangling bonds associated with the absence of hydrogen passivation. However, when graphene is used in an electronic device, the graphene layer is usually deposited on a surface of the SiO_2 _substrate where the atomic structure of the surface is usually considered to reconstruct to eliminate the dangling bonds [24]. In addition, the atomic force microscopy [AFM] image indicated that the height of a single graphene layer on the SiO_2 _substrate is around 4 Å, which is a bit larger than the 3.4 Å of the graphite interlayer distance [25]. Therefore, the interaction between graphene and the SiO_2 _surface should be a weak van der Waals interaction without the formation of strong covalent bonds.

In this work, we investigate the graphene/α-SiO_2_(0001) interface through density functional theory [DFT] calculations with considerations on surface reconstruction of SiO_2_. The interfacial atomic structure and the effects of the substrate on corresponding electronic structure of graphene will also be studied.

### Simulation details

There are two fundamental types of surfaces: the surface with the termination of the Si atoms or the surface with the termination of the O atoms. Based on the atomic structure study of α-SiO_2_(0001) surface using the first principles [24], the O-terminated surface was more stable where covalent bonds between two O atoms on a particular Si atom were formed. Therefore, the O-terminated surface is selected for study in this work.

**Figure 1:**
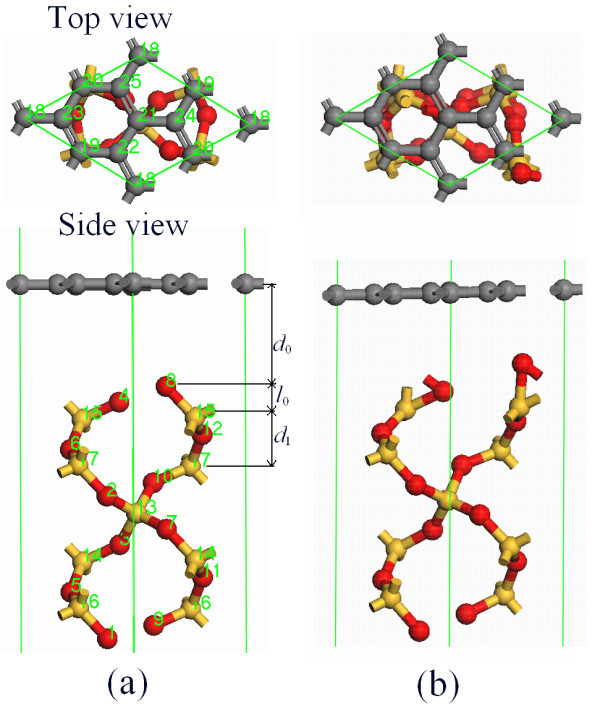
**Atomic stacking sequence of graphene/α-SiO_2_(0001) interface without relaxation (a) and in structure A (b)**. The yellow, gray, and red spheres show Si, C, and O atoms. The numbers are the index of atoms in the simulation. The meanings of parameters in the figure are given in the text.

All DFT calculations in this work are implemented by the DMOL^3 ^code, which utilizes a norm-conserving pseudopotential to perform the first principle quantum mechanical calculations [26]. Local density approximation with the PWC function is employed as exchange correlation function [27]. The spin restriction is taken, which would not lead to a big error since the dangling oxygen bonds would be eliminated through the formation of covalent bonds between the two O atoms on the particular Si atom as shown in Figure 1b[24]. The *K *points are 6 × 6 × 6 for the bulk and 6 × 6 × 1 for all slabs, which make the convergence tolerance of energy of 1.0 × 10^-5 ^eV/atom, maximal force of 0.03 eV/Å, and displacement of 1.0 × 10^-3 ^Å.

The graphene/α-SiO_2_(0001) interface is constructed based on the bulk structures. A vacuum thickness of 12 Å is added above the graphene layer to ensure the interaction between the repeated slabs in the normal direction of the surface to be small enough. The simulation cell parameters of the graphene and the SiO_2 _substrate are *a *= *b *= 4.920 Å, *α *= *β *= 90°, and *γ *= 120° and *a *= *b *= 4.910 Å, *α *= *β *= 90°, and *γ *= 120°, respectively, where a small lattice mismatch is within a range of 0.3%, as shown in Figure 1a. Based on the AFM result, it is known that the height of the single graphene layer on the SiO2 substrate is around 4 Å [25]. Thus, in the initial structure of graphene/α-SiO2 interface, as shown in Figure 1a, the distance between graphene and SiO2 [d0] is set as 4.0 Å. In the simulation process, a structural relaxation process is allowed for C atoms in the graphene and all Si and O atoms in the substrate, except that the atoms of the lowest O-Si-O monolayer are fixed.

## Results and discussion

The atomic structure of the favorite graphene/α-SiO_2_(0001) interface configuration with the distance between the graphene and SiO_2 _surface, *d*_0 _= 2.820 Å, is shown in Figure 1b, which is named as structure A; *d*_0 _is in the van der Waals distance range, but it is smaller than the interlayer distance of graphite of 3.4 Å. From the figure, the surface reconstruction occurred after the relaxation which a covalent bond forms between the two O atoms on the particular Si atom to eliminate the dangling bonds. The related structure parameters are given in Table 1. As shown in the table, a bond length expansion between the surface or interface atoms is found with *l*_0bulk _<*l*_0A _<*l*_0clean_, where *l*_0 _denotes Si-O bond length, and the subscripts bulk, clean, and A denote bulk SiO_2_, clean SiO_2 _slab, and structure A, respectively. The result *l*_0bulk _= 1.596 Å is consistent with the reported result of 1.61 Å [20] while the extension of Si-O bonds at the surface or interface is found. We consider that the extension is caused by the one Si-O broken bond on the substrate surface or at the interface. In addition, the weak interaction between the graphene and the SiO_2 _surface slightly reduces the *l*_0 _at the interface compared with that on the surface.

**Table 1:** Atomic structure parameters of bulk SiO_2_, clean SiO_2_(0001) surface, and graphene/SiO_2_(0001) interface

Measurement	Bulk SiO_2_	Clean SiO_2 _slab	Graphene/SiO_2 _(structure A)
*d*_0 _(Å)			2.820
*d*_1 _(Å)	1.798	1.685	1.619
*l*_0 _(Å)	1.596	1.620	1.616
*α*_1 _(°)	146.529	137.140	135.932
*l*_C-C _(Å)			1.430

*d*_1 _is the distance between the first and the second Si atom layers; *l*_0 _is the length of Si-O bond on the surface or interface; *α*_1 _is the value of the Si-O-Si angle; *l*_C-C _is the average bond length of C-C.

In addition, Table 1 also shows the distance between the first and second Si layers [*d*_1_] and the Si-O-Si bond angle [*α*_1_] in different structures; *d*_1bulk _>*d*_1clean _>*d*_1A _and *α*_1bulk _>*α*_1clean _>*α*_1A _are found. In light of Figure 1, *d*_1 _is affected by both *l*_0 _and *α*_1_. The former brings out *d*_1bulk _<*d*_1clean_, and the latter leads to *d*_1bulk _>*d*_1clean_. Therefore, the effect of *α*_1 _on *d*_1 _is stronger than that of *l*_0_. Comparing structure A with the clean SiO_2 _surface slab, both *l*_0 _and *α*_1 _lead to the decrease of *d*_1_, where *d*_1 _and *l*_0 _of structure A shrink to about 4%.

Quantitative experimental data for the interaction strength of graphene/substrate interface is very limited. The first principle calculations showed that the binding energy of graphene on a Si-terminated SiO_2 _surface is around 20 meV/C atom with interface distance *d*_0 _= 3.29 Å and that on a hydrogen passivation O-terminated SiO_2 _surface is 0.13 eV/C atom with *d*_0 _= 2.58 Å [20]. In addition, the interlayer binding energy in graphite was reported to be 50 to 60 meV [28]. In the system of structure A, the binding energy calculated between graphene and the SiO2 substrate is about 77 meV/C atom, which is larger than that in graphite and also in the graphene on Si-terminated SiO_2 _surface, but it is smaller than that on the hydrogen-passivated O-terminated SiO_2 _surface. Note that *d*_0 _in structure A is 2.820 Å, which is smaller than 3.14 Å in graphite and graphene laid on the Si-terminated SiO_2 _surface, but it is larger than that on hydrogen-passivated O-terminated SiO_2 _surface. It is known that the binding energy is inversely proportional to *d*_0_. The system of graphene on hydrogen-passivated O-terminated SiO_2 _surface has the smallest *d*_0 _and the strongest binding energy. A previous study implied that the C-C bond is weakened through the strengthening of bonds to the substrate [29]. A similar phenomenon is found in structure A where the C-C bond length of graphene is 1.430 Å, which is longer than the 1.420 Å in graphite. Thus, the adsorption of graphene on SiO_2 _with structure A is stronger than that between the bulk graphite layers as shown previously.

**Figure 2:**
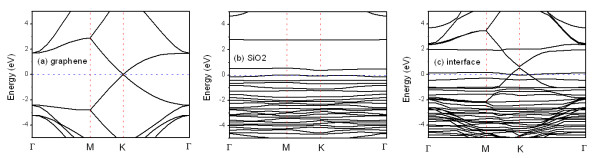
**Calculated band structures for graphene monolayer (a), clean α-SiO_2_(0001) slab, (b) and graphene/α-SiO_2 _interface (c)**. The dash line at 0 value denotes the Fermi level.

On the other hand, in order to understand the effect of substrate on the graphene electrical properties, the band structure for three different systems in Figure 1b, namely free graphene monolayer, clean SiO_2_(0001) slab, and graphene/SiO_2 _interface, are represented in Figure 2. The band structure of free graphene monolayer is calculated on a periodic structure where the graphene monolayer is separated by the same vacuum distance as that for the graphene/SiO_2 _slabs. This band structure shows the crossing of π and π* bands at the *K *point and also at the Fermi level, which agrees with a well-known result that graphene is semimetallic with a 0 bandgap. The band structure of the interface shows that the bands of graphene layer are open with a 0.13-eV gap at the *K *point. It is interesting to note that the Fermi level is lowered with the amount of transferred charges. Thus, the charge should be transferred from graphene to the substrate in structure A. As a consequence of the charge transfer, the SiO_2 _substrate induces *p*-type doping in the graphene. Except for these variations, the band structure of interface is almost identical with the sum of band structure of the free graphene monolayer and SiO_2 _slab, as shown in Figure 2a, b.

The charge transfer and atomic charge can be obtained using the Mulliken analysis; it is shown in Table 2. Mulliken analysis is performed using a projection of the plane wave states onto a localized basis with the technique described by Sanchez-Portal et al. [30]. Subsequently, the resulting projected states are performed using the Mulliken formalism [31]. This technique has been widely used to analyze the electronic structures performed with linear combinations of atomic orbital basis sets. As shown in Table 2, all C atoms of graphene are positively charged in structure A, and the graphene transfers 0.144 *e *to the SiO_2 _substrate where *e *denotes one electron charge. This is consistent with the result of the band structure where the *p*-type-doped graphene induced by the substrate is found.

**Table 2:** Charge transfer and atomic charge obtained by Mulliken analysis

Atom index	In bulk SiO_2 _and graphite (*e*)	In clean SiO_2 _slab and graphene (*e*)	In structure A (*e*)
O1	-0.781	-0.347	-0.395
O2	-0.781	-0.791	-0.916
O3	-0.781	-0.781	-0.908
O4	-0.781	-0.396	-0.448
O5	-0.781	-0.747	-0.870
O6	-0.781	-0.778	-0.890
O7	-0.781	-0.800	-0.929
O8	-0.781	-0.293	-0.424
O9	-0.781	-0.444	-0.485
O10	-0.781	-0.837	-0.955
O11	-0.781	-0.797	-0.911
O12	-0.781	-0.746	-0.877
Si13	1.562	1.582	1.834
Si14	1.562	1.587	1.843
Si15	1.562	1.510	1.681
Si16	1.562	1.463	1.643
Si17	1.562	1.605	1.863
C18	0	0	0.017
C19	0	0	0.023
C20	0	0	0.014
C21	0	0	0.018
C22	0	0	0.016
C23	0	0	0.010
C24	0	0	0.020
C25	0	0	0.026
*Q*			0.144

Charges of atoms in bulk SiO_2 _and graphite, clean SiO_2_(0001) slab and free graphene layer, and graphene/SiO_2_(0001) interface with structure A, as well as charge transfer (*Q*) between graphene and the SiO_2_(0001) substrate obtained by Mulliken analysis; *e *denotes one electron charge.

In addition, the electron charges of O4 and O8 atoms at the SiO_2 _surface are -0.448 and -0.424 *e*, respectively, for structure A. Those in the clean SiO_2_(0001) slab are -0.396 and -0.293 *e*, and in bulk SiO_2 _are both -0.781 *e*. On the other hand, the charges of Si15, which binds with O4 and O8, are 1.681, 1.510, and 1.562 *e *in structure A, clean SiO_2 _slab, and bulk SiO_2_, respectively. It is known that the bond strength is in proportion with the multiplication absolute value of changes of the O and Si atoms. Therefore, the interaction between O4 and Si15, and O8 and Si15 are the strongest in bulk SiO_2_, followed by that in structure A, and the weakest was that in the SiO_2 _slab. These also agree with the results in Table 1 where the Si-O *l*_0 _at the surface or interface is *l*_0bulk _<*l*_0A _<*l*_0clean_.

## Conclusions

In conclusion, DFT calculations were employed to study the graphene/α-SiO_2_(0001) interface with an oxygen-terminated SiO_2 _surface. After geometry relaxation, a stable structure with a distance between graphene and the SiO_2 _surface, *d*_0 _= 2.805 Å, was observed. In addition, the structure reconstruction of the SiO_2 _surface took place where the two O atoms binding on a particular Si atom form a covalent bond on the surface to eliminate the surface dangling bonds. Simulation results indicate that the interface interaction is 77 meV/C atom, which indicates that the van der Waals force dominates the interaction. Furthermore, the SiO_2 _substrate induces *p*-type doping in graphene and open a small gap of 0.13 eV at the Dirac point of graphene.

## Competing interests

The authors declare that they have no competing interests.

## Authors' contributions

ZA did the calculation and drafted the manuscript. ZW, MJ, and SL co-drafted the manuscript. All authors read and approved the final manuscript.
